# LL-37 as a Powerful Molecular Tool for Boosting the Performance of Ex Vivo-Produced Human Dendritic Cells for Cancer Immunotherapy

**DOI:** 10.3390/pharmaceutics14122747

**Published:** 2022-12-08

**Authors:** Dmitry Stakheev, Pavla Taborska, Katerina Kalkusova, Jirina Bartunkova, Daniel Smrz

**Affiliations:** Department of Immunology, Second Faculty of Medicine, Charles University and Motol University Hospital, 150 06 Prague, Czech Republic

**Keywords:** LL-37, dendritic cells, CD8^+^ T cells, cellular immunotherapy, cancer

## Abstract

Ex vivo-produced dendritic cells (DCs) constitute the core of active cellular immunotherapy (ACI) for cancer treatment. After many disappointments in clinical trials, the current protocols for their preparation are attempting to boost their therapeutic efficacy by enhancing their functionality towards Th1 response and capability to induce the expansion of cytotoxic tumor-specific CD8^+^ T cells. LL-37 is an antimicrobial peptide with strong immunomodulatory potential. This potential was previously found to either enhance or suppress the desired anti-tumor DC functionality when used at different phases of their ex vivo production. In this work, we show that LL-37 can be implemented during the whole process of DC production in a way that allows LL-37 to enhance the anti-tumor functionality of produced DCs. We found that the supplementation of LL-37 during the differentiation of monocyte-derived DCs showed only a tendency to enhance their in vitro-induced lymphocyte enrichment with CD8^+^ T cells. The supplementation of LL-37 also during the process of DC antigen loading (pulsation) and maturation significantly enhanced the cell culture enrichment with CD8^+^ T cells. Moreover, this enrichment was also associated with the downregulated expression of PD-1 in CD8^+^ T cells, significantly higher frequency of tumor cell-reactive CD8^+^ T cells, and superior in vitro cytotoxicity against tumor cells. These data showed that LL-37 implementation into the whole process of the ex vivo production of DCs could significantly boost their anti-tumor performance in ACI.

## 1. Introduction

Active cellular immunotherapy (ACI) (also termed “cancer vaccines”) is a modern and quickly developing anti-tumor immunotherapeutic approach [1]. The ACI is based on ex vivo-produced antigen-presenting cells (APCs) that elicit a targeted immune response in vivo [1]. Researchers have developed multiple ways to produce APCs ex vivo, namely, dendritic cells (DCs), which constitute the core of current ACI [2,3]. DCs can be differentiated ex vivo from hematopoietic stem cells or monocytes [4,5]. Because monocytes can be readily isolated from peripheral blood and their differentiation requires only several days in cell culture in the presence of selected cytokines, these cells have often been used for ACI in clinical trials [6]. Although the evidence of the past several years has shown that DC therapeutic efficacy is evident in many cancers and thus proves ACI’s concept, it is still far from satisfactory [7]. The accumulated experience has pointed to the fact that even though the contemporarily produced DCs can promote anti-tumor immune responses [8,9,10], this promotion is weak and presumably too dependent on the functional immune system of the patient. In late-stage cancer, this functionality is eroded and may even impact the ex vivo production of DCs [11]. To challenge this suppression, DC production needs to be much more robust, and DC activity needs to be much better at activating the cytotoxic CD8^+^ T cells, which can immediately eliminate cancer cells to decrease the tumor burden and relieve the patient’s immune system from immunosuppression. However, the protocols and molecular tools for the robust production of such DCs are missing.

One previously investigated molecular tool for improving DC production is LL-37. LL-37 belongs to antimicrobial peptides [12] and is expressed by cells that encounter pathogens, such as skin and intestine epithelial cells [13]. LL-37 is one of the key components of the innate immune system and can act against pathogens or cancer cells [12]. In vitro, the LL-37 treatment of human peripheral blood mononuclear cells (PBMCs) can promote the generation of regulatory T cells and decrease the expression of pro-inflammatory cytokines [14]. LL-37 can also impair DC functionality once used during their maturation [15]. On the other hand, LL-37 supplementation during the DC differentiation was found to promote the pro-inflammatory cytokine production and Th1 polarization of DC-induced T cells [16]. A recent study also showed that LL-37 supplementation during DC differentiation enhanced the anti-tumor activity of bone-marrow-derived DCs [17]. In addition, this study also showed that human-monocyte-derived DCs differentiated in the presence of LL-37 had enhanced potential for inducing the proliferation of autologous CD8^+^ T cells [17]. Therefore, LL-37 can display ambivalent contributions to the DC functionality, depending on how it is used during the process of DC production.

In the present study, we implemented LL-37 during the whole process of the ex vivo production of human-monocyte-derived DCs. We found that not only the DC differentiation but concomitantly also DC pulsation and maturation in the presence of LL-37 were important for DCs to substantially enhance the production of tumor-specific CD8^+^ T cells and that this production was also associated with the downregulated expression of PD-1. Notably, whereas DC-expanded lymphocytes were able to stop the expansion of cultured tumor cells, the (LL-37-treated DC) expanded lymphocytes could even cause a contraction of the expanding tumor cell cultures. Thus, LL-37 represents a powerful molecular tool for boosting the performance of ex vivo-produced human-monocyte-derived DCs for cancer immunotherapy.

## 2. Materials and Methods

### 2.1. Specimens

The buffy coats obtained from healthy blood donors were from the Institute of Hematology and Blood Transfusion in Prague. The blood donors signed written informed consent to provide buffy coats for research. The institutional research committee approved all the experimental protocols (the Ethics Committee of the University Hospital Motol in Prague (protocol code: EK—753.1.8/21)). The experiments were performed in accordance with the 1964 Declaration of Helsinki and its later amendments or comparable ethical standards.

### 2.2. Preparation of Immature-Monocyte-Derived DCs

Peripheral blood mononuclear cells (PBMCs) were prepared from the buffy coats of healthy blood donors using a density gradient separation as described previously [18]. PBMCs were then cryopreserved in the human-plasma- and DMSO-containing medium (RPMI 1640 medium (Thermo Scientific, Waltham, MA, USA), 10% DMSO (Sigma-Aldrich, St. Louis, MO, USA), 10% human plasma serum (One Lambda, Canoga Park, CA, USA), 100 U/mL penicillin–streptomycin, 2 mM GlutaMax, 1 mM nonessential amino acid mix, and 1 mM sodium pyruvate (Thermo Scientific)).

The cryopreserved PBMCs were reconstituted in a fetal-bovine-serum-containing medium (KM medium; RPMI 1640 medium (Thermo Scientific), 10% heat-inactivated fetal bovine serum (HyClone, GE Healthcare Life Sciences, South Logan, UT, USA), 100 U/mL penicillin–streptomycin, and 2 mM GlutaMax (Thermo Scientific)). The cells were transferred to a tissue culture flask (TPP, Trasadingen, Switzerland) and were incubated for 2 h at 37 °C and 5% CO_2_. The non-adherent fraction (lymphocytes) was removed and cryopreserved in the human-plasma- and DMSO-containing medium. The flasks were rinsed twice with a phosphate-buffered saline (PBS). The adherent cells (monocytes) in the flasks were then cultured in KM medium supplemented with 1000 IU/mL of the granulocyte-macrophage-colony-stimulating factor (GM-CSF) and 1000 IU/mL of IL-4 (Immunotools, Friesoythe, Germany). The cell cultures were, or were not, supplemented with 2.5 μg/mL of LL-37 (cat no. 94261, Sigma-Aldrich). On day 4 of the cell culture, the cells were supplemented with 1000 IU/mL of GM-CSF and IL-4. On day 5 of the cell culture, the cells were harvested, pelleted by centrifugation, and resuspended at a 1 × 10^6^ cells/mL concentration in the fresh KM medium with 1000 IU/mL of GM-CSF and IL-4.

### 2.3. Pulsation and Maturation of Immature-Monocyte-Derived DCs

The lysate for pulsation was prepared from trypsinized PC-3 cells [19], which were previously cultured under adherent conditions in the KM medium. The trypsinized PC-3 cells were resuspended in KM medium at a 1 × 10^6^ cells/mL concentration and were lysed by two cycles of freezing and thawing. The lysates were inactivated by 2 cycles of UV irradiation with a Bio-Link BLX E312 crosslinker (Vilber Lourmat, Collégien, France) for 10 min at RT (312 nm, 2.55 J/cm^2^). The lysate was, or was not, then supplemented with 5 μg/mL of LL-37 and incubated for 20 min at RT. After incubation, 500 μL of the PC-3 cell lysate was combined with 500 μL suspension of DCs (1 × 10^6^ cells/mL) in a flat-bottom 48-well plate well (Nalgene, Rochester, NY, USA) and incubated (37 °C, 5% CO_2_) for 4 h. The cells were then supplemented with 10 μg/mL of a TLR7/8 agonist, R-848 (Enzo Life Sciences, Farmingdale, NY, USA), and allowed to mature by overnight incubation (37 °C, 5% CO_2_, 18–24 h).

### 2.4. DC Maturation Analysis

The matured DCs were harvested and transferred to a V-bottom 96-well plate (Nalgene). The cells were pelleted, washed with ice-cold PBS supplemented with 2 mM EDTA (PBS/EDTA), and stained with the following fluorescent-labeled antibodies: CD83-FITC, CD86-PE (Beckman Coulter, Brea, CA, USA), or HLA-DR-Pe-Cy7 (Becton Dickinson, Franklin Lakes, NJ, USA). After 30–60 min incubation at 4 °C in the dark, the cells were pelleted and rinsed twice with ice-cold PBS/EDTA. The cells were resuspended, transferred to 5-mL round-bottom FACS tubes (Nalgene), supplemented with 100 ng/mL of DAPI (Thermo Scientific), and immediately analyzed using FACSAria II or a BD LSRFortessa flow cytometer (Becton Dickinson). The FlowJo software (Tree Star, Ashland, OR, USA) was used to analyze the acquired flow cytometry data.

### 2.5. DC-Induced Stimulation of Autologous Lymphocytes

The autologous lymphocytes for stimulation with DCs were, with minor changes, prepared as previously described [20]. Briefly, the cryopreserved autologous lymphocytes were reconstituted in a human-plasma-containing medium (LM medium; RPMI 1640 medium, 5% human plasma serum (One Lambda), 2 mM GlutaMax, 100 U/mL penicillin–streptomycin, 1 mM nonessential amino acid mix, and 1 mM sodium pyruvate (Thermo Scientific)). The cells were then cultured overnight (37 °C, 5% CO_2_, 18–24 h). The next day, the reconstituted autologous lymphocytes were pelleted and resuspended in the LM medium at the concentration 1 × 10^6^ cells/mL, and 1 mL was transferred to a flat-bottom 48-well plate well (Nalgene). The PC-3 lysate-pulsed and matured DCs were pelleted and resuspended in the LM medium at 1 × 10^6^ cells/mL. A 200 μL volume of the suspension was combined in the flat-bottom 48-well plate well with the 1 mL of autologous lymphocytes (5:1 ratio of the autologous lymphocytes and DCs). The cells were cultured for 14 days (37 °C, 5% CO_2_). The cells were supplemented with IL-2 (20 IU/mL; PeproTech, Rocky Hill, NJ, USA) every 2–3 days of the cell culture. At the same time interval, a fresh LM medium was added to the cell culture with or without prior hemi-depletion of the cell culture supernatant.

To determine the expression of TIM-3 and PD-1 on DC-stimulated autologous lymphocytes, the cells were transferred to a V-bottom 96-well plate (Nalgene), pelleted, rinsed with ice-cold PBS/EDTA, and stained with the following antibodies: CD3-PerCP-Cy5.5, CD4-PE-Cy7 (eBiosciences, San Diego, CA, USA), CD8-Alexa Fluor 700 (Exbio, Prague, Czech Republic), TIM-3-PE, and PD-1-APC (BioLegend, San Diego, CA, USA) for 30–60 min at 4 °C. The cells were pelleted and rinsed with ice-cold PBS/EDTA. The cells were resuspended with PBS/EDTA containing 100 ng/mL of DAPI (Thermo Scientific). The cells were immediately analyzed using flow cytometers, and the FlowJo software was used to analyze the data.

### 2.6. Determination of the Tumor-Cell-Reactive T Cells in Cultured DC-Induced Autologous Lymphocytes

The 14-day-cultured, DC-stimulated lymphocytes were re-stimulated with live PC-3 cells at the ratio of 5:1 (lymphocytes: PC-3 cells) and were cultured (37 °C, 5% CO_2_) for 5 h in the presence of brefeldin A (BioLegend) and supplemented 1 h after the stimulation. The samples stimulated with vehicle alone (LM medium) were used as the control. After the stimulation, the cells were stained with a fixable live/dead stain, fixed, and permeabilized as described [20]. The cells were then stained with the above-described fluorescent-tagged CD3-, CD4-, and CD8-specific antibodies and with fluorescent-tagged IFN-γ- (PE) and TNF-α (APC) (Becton Dickinson)-specific antibodies. The stained cells were analyzed using the above-stated flow cytometers, and the acquired data were analyzed by FlowJo. The percentage of the PC3-reactive IFN-γ- and/or TNF-α-producing T-cell populations was calculated as the difference between the percentages of the IFN-γ- and/or TNF-α-producing T cells in control- and PC-3-stimulated samples.

### 2.7. Evaluation of the Tumor Cell Co-Culture with DC-Induced Autologous Lymphocytes

TagFP635-PC-3 cells were prepared as described previously [21]. The 50 × 10^3^ TagFP635-PC-3 cells in 1 mL of KM medium were seeded in a flat-bottom 48-well plate well (Nalgene) and cultured for 2 days at 37 °C in 5% CO_2_. The supernatant was removed, and 1 × 10^6^ of the 14-day-cultured, DC-stimulated lymphocytes were added to the 2-day cultures of the TagFP635-PC-3 cells in the flat-bottom 48-well plate well. The cells were allowed to sediment for 10 min, and then the mean fluorescence intensity (MFI) of TagFP635-PC-3 cells in the wells was determined. The cells were then co-cultured for 7 days at 37 °C in 5% CO_2_, and the MFI of the TagFP635-PC-3 cells in the cell co-culture was determined on days 2, 5, and 7.

### 2.8. Statistical Analysis

The values were calculated from the sample size (*n*) using GraphPad Prism 6 (GraphPad Software, La Jolla, CA, USA). A paired two-tailed Student’s *t*-test was used to calculate the statistical significance between the two variables. One-way ANOVA or repeated measures of one-way ANOVA with Tukey’s post-test was used to determine the statistical significance among more than two of the variables. The statistical significance was indicated as follows: ^NS^ *p >* 0.05, * *p* < 0.05, ** *p* < 0.01, *** *p* < 0.001. Biorender.com was used to produce graphical images (agreement number: LL24QM9QRU).

## 3. Results

### 3.1. LL-37 Was Important for Both DC Differentiation and Pulsation to Enhance DC-Mediated Expansion of CD8^+^ T Cells with Downregulated Expression of PD-1

**Figure 1:**
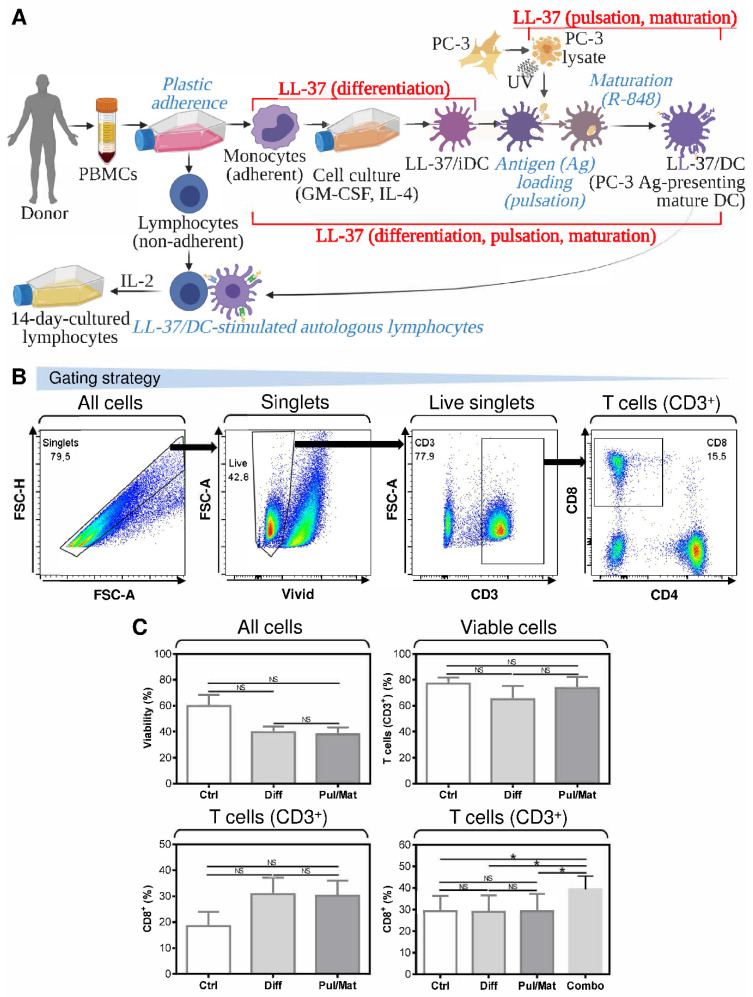
The impact of LL-37 on the DC functionality when implemented during DC differentiation or pulsation and maturation. (**A**) The LL-37 treatment strategy during ex vivo production of monocyte-derived DCs. The DCs were first differentiated from PBMCs to immature DCs in the presence of LL-37 (LL-37/iDC); then, following pulsation with antigen (Ag), they were matured using TLR7/8 agonist R-848 (LL-37/DC). The functionality of LL-37/DCs was tested in the 14-day-cultured lymphocytes generated by the stimulation of autologous lymphocytes with LL-37/DCs and subsequent cell culturing in the presence of a low concentration IL-2 (20 IU/mL). (**B**) The gating strategy of flow cytometry data obtained from analyses of DC-stimulated and 14-day-cultured autologous lymphocytes. (**C**) The viability (top left panel), frequency of T cells (CD3^+^; top right panel), and their CD8^+^ population (bottom panels) in the 14-day-cultured autologous lymphocytes stimulated with DCs treated with LL-37 during their differentiation (Diff), or PC-3 pulsation and R-848-induced maturation (Pul/Mat), or differentiation, PC-3 pulsation, and R-848-induced maturation (Combo). In (**C**)**,** mean + SEM and significances of differences among the groups are indicated (^NS^ *p* > 0.05, * *p* < 0.05; (**C**, top panels and bottom left panel) Ctrl: *n* = seven donors, Diff and Pul/Mat: *n* = six donors; one-way ANOVA with Tukey’s post-test; (**C**, bottom right panel) *n* = six donors; repeated measures (paired) one-way ANOVA with Tukey’s post-test).

To produce monocyte-derived DCs for cancer immunotherapy ex vivo, peripheral blood monocytes are first differentiated into immature DCs. These immature DCs are then loaded (pulsed) with tumor antigens and matured with maturation cocktails [22]. To investigate the impact of LL-37 on DC production, we first supplemented LL-37 during the DC differentiation or pulsation and maturation (Figure 1A). The impact of LL-37 on DC functional phenotypes was then evaluated through the DC-mediated enrichment of cultured autologous lymphocytes with T cells and their cytotoxic CD8^+^ population (Figure 1B). The source of tumor antigens was a UV-inactivated lysate of the human prostate cancer cell line PC-3 [19]. As shown in Figure 1C, regardless of the LL-37 treatment strategy, LL-37 did not significantly affect the viability of DC-stimulated autologous lymphocytes (Figure 1C, top left panel) nor change the frequency of T cells in these lymphocytes (Figure 1C, top right panel). The frequency of CD8^+^ T cells was also not significantly affected by the LL-37 treatments once LL-37 was supplemented during either DC differentiation or their pulsation and maturation (Figure 1C, bottom left panel). However, the CD8^+^ T cell frequency was significantly increased when the LL-37 treatment was implemented during both DC differentiation and their pulsation and maturation (Figure 1C, bottom right panel).

**Figure 2:**
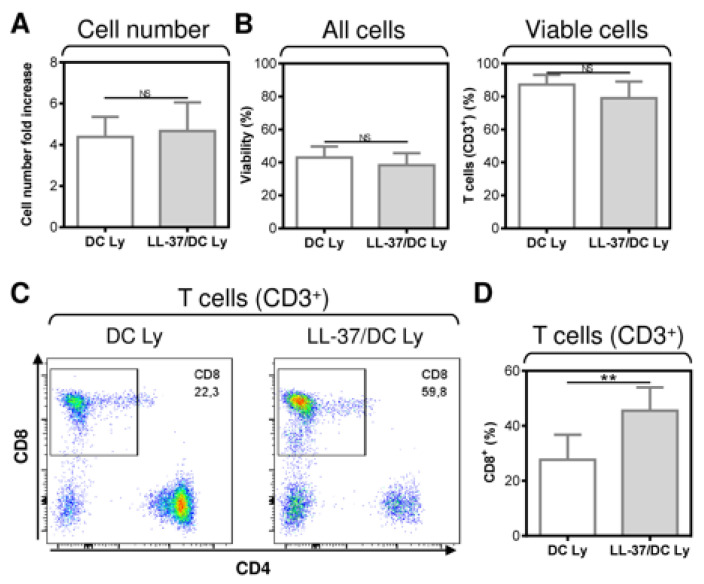
The application of LL-37 during DC production (differentiation, pulsation, and maturation) significantly enhanced CD8^+^ T cell enrichment of DC-stimulated autologous lymphocytes. (**A**) The cell number fold increase in the 14-day autologous lymphocytes stimulated with DCs (DC Ly), or DCs produced in the presence (differentiation, pulsation, and maturation) of LL-37 (LL-37/DC Ly). (**B**–**D**) The DC-stimulated and 14-day-cultured autologous lymphocytes were analyzed by flow cytometry. The gating strategy was as in Figure 1B, and cell viability (**B**, left panel), the frequency of T cells (CD3^+^) (**B**, right panel), and their CD8^+^ population (**C**,**D**) were determined. In (**A**,**B**,**D**), mean + SEM and significances of differences between the group of DC Ly and LL-37/DC Ly samples are indicated (^NS^ *p* > 0.05, ** *p* < 0.01; *n* = eight donors; paired two-tailed Student’s *t*-test).

The data indicated that LL-37 impacted DC functionality when LL-37 was present during both DC differentiation, pulsation, and maturation. We next investigated whether the combined LL-37 treatments affected other parameters of the DC-stimulated autologous lymphocytes. As shown, the expansion (Figure 2A), viability (Figure 2B, left panel), and T cell frequency (Figure 2B, right panel) of the lymphocytes were not affected by the combined LL-37 treatments. However, consistent with the results in Figure 1C, the combined LL-37 treatments produced a significant enhancement in the proportions of CD8^+^ T cells in the DC-stimulated lymphocytes (Figure 2C,D).

**Figure 3:**
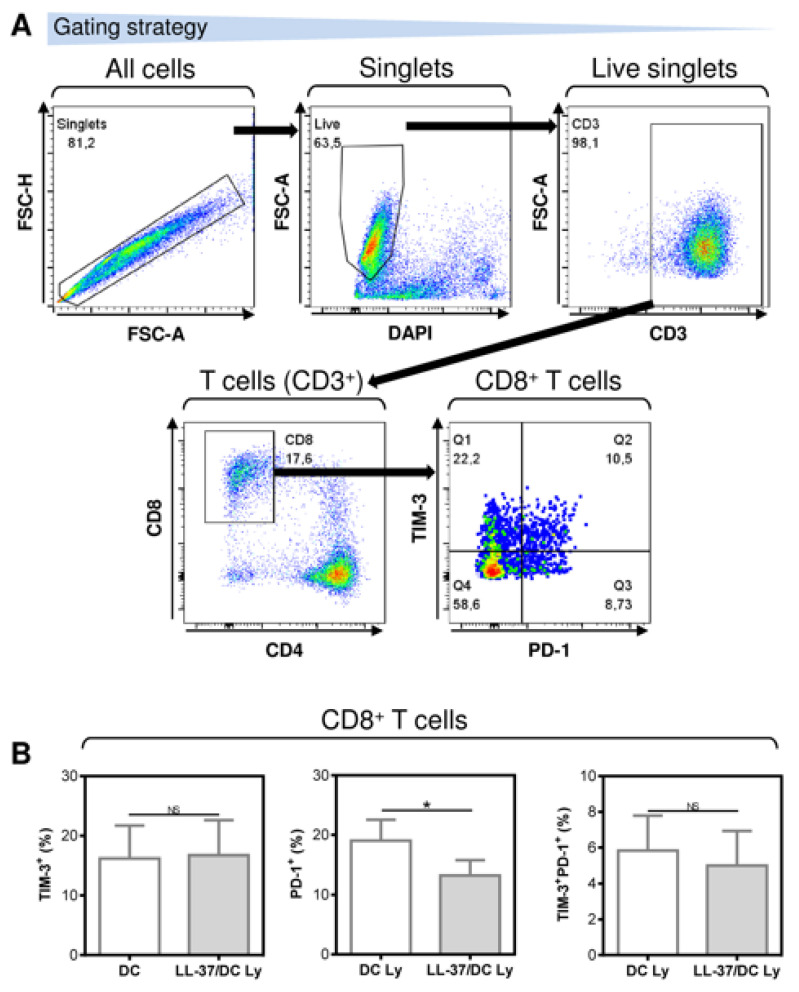
The application of LL-37 during DC production (differentiation, pulsation, and maturation) significantly downregulated the surface expression of PD-1 but not TIM-3 in CD8^+^ T cells of DC-stimulated autologous lymphocytes. (**A**) The gating strategy of flow cytometry data. (**B**) The expression of TIM-3, PD-1, and TIM-3 together with PD-1 in the 14-day autologous lymphocytes stimulated with DCs (DC Ly), or DCs produced in the presence (differentiation, pulsation, and maturation) of LL-37 (LL-37/DC Ly). In (**B**), mean + SEM and significances of differences between the group of DC Ly and LL-37/DC Ly samples are indicated (^NS^ *p >* 0.05, * *p* < 0.05; *n* = six donors; paired two-tailed Student’s *t*-test).

NK cells and T cells are often associated with the increased expression of inhibitory molecules PD-1 and TIM-3, whose blockade with anti-PD-1 and/or anti-TIM-3 blocking antibodies improves the functionality of these cells [23,24,25]. Our analyses revealed that LL-37 significantly decreased the frequency of PD-1-expressing CD8^+^ T cells in the DC-stimulated lymphocytes. These CD8^+^ T cells had a lower surface expression of the checkpoint inhibitor molecule PD-1 (Figure 3). On the other hand, no impact of LL-37 was observed on the surface expression of the immune checkpoint molecule TIM-3 (Figure 3). This finding showed that LL-37 also promoted DC-mediated cell culture enrichment with PD-1-downregulated CD8^+^ T cells, which could also contribute to the increased resistance of these cells to PD-1-mediated immunosuppression.

### 3.2. LL-37 Was Important for DC Differentiation, Maturation, and Pulsation to Enhance DC-Mediated Expansion of Tumor-Cell-Specific CD8^+^ T Cells

**Figure 4:**
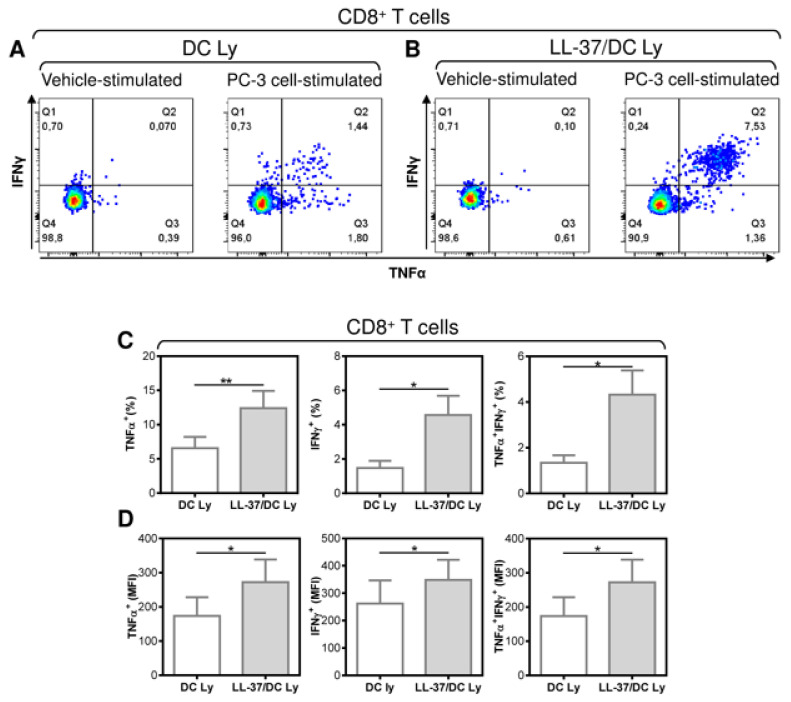
The application of LL-37 during DC production (differentiation, pulsation, and maturation) significantly enhanced the enrichment of DC-stimulated autologous lymphocytes with tumor-cell-specific CD8^+^ T cells. The 14-day autologous lymphocytes stimulated with DCs (DC Ly) or DCs produced in the presence (differentiation, pulsation, and maturation) of LL-37 (LL-37/DC Ly) were re-stimulated with live PC-3 cells at the ratio of 5:1 (lymphocytes/PC-3 cells), and the frequency of TNF-α-, IFN-γ-, and TNF-α/IFN-γ-producing CD8^+^ T cells was determined by intracellular cytokine staining using flow cytometry. (**A**,**B**) The gating strategy of CD8^+^ T cells was as in Figure 1B and was followed by the gating of the cytokine-producing CD8^+^ T cells in vehicle- (vehicle-stimulated) or PC-3 cell (PC-3 cell-stimulated)-stimulated DC Ly (**A**) and LL-37/DC Ly (**B**) samples. (**C**) The frequency of TNF-α- (left panel), IFN-γ- (middle panel), and TNF-α/IFN-γ (right panel)-producing CD8^+^ T cells was determined by intracellular cytokine staining in DC Ly- and LL-37/DC Ly samples. (**D**) The mean fluorescence intensity (MFI) of TNF-α- (left panel), IFN-γ- (middle panel), and TNF-α/IFN-γ (right panel)-producing CD8^+^ T cells was determined by intracellular cytokine staining in DC Ly and LL-37/DC Ly samples. In (**C**,**D**), mean + SEM and significances of differences between the group of DC Ly and LL-37/DC Ly samples are indicated (* *p* < 0.05, ** *p* < 0.01; (**C**) *n* = eight donors, (**D**) *n* = six donors; paired two-tailed Student’s *t*-test).

The necessity to treat DCs with LL-37 during their differentiation, pulsation and maturation to attain a significant enhancement of the DC-mediated expansion of CD8^+^ T cells indicates that this expansion could also be associated with the promotion of CD8^+^ T cells that are reactive to tumor cells expressing the pulsed antigen. Therefore, we next investigated whether the DC-expanded CD8^+^ T cells were reactive to cultured PC-3 cells, the lysate of which was used for the pulsation, and whether the proportions of these PC-3-reactive T cells were enhanced after their treatment with IL-37. The reactivity of the T cell populations to PC-3 cells was evaluated through intracellular staining for TNF-α and IFN-γ in the PC-3-stimulated T cells (Figure 4A,B). As shown, LL-37 enhanced the proportions of TNF-α-, IFN-γ-, and TNF-α/IFN-γ-producing CD8^+^ T cells that were reactive to PC-3 cells (Figure 4C). LL-37 also enhanced the strength of the cytokine production as determined through the intensities of their staining (Figure 4D). These data showed that LL-37 also enhanced proportions of CD8^+^ T cells that were reactive to cells expressing the pulsed antigens.

### 3.3. LL-37 Did Not Enhance the Maturation of the Antigen-Pulsed DCs

**Figure 5:**
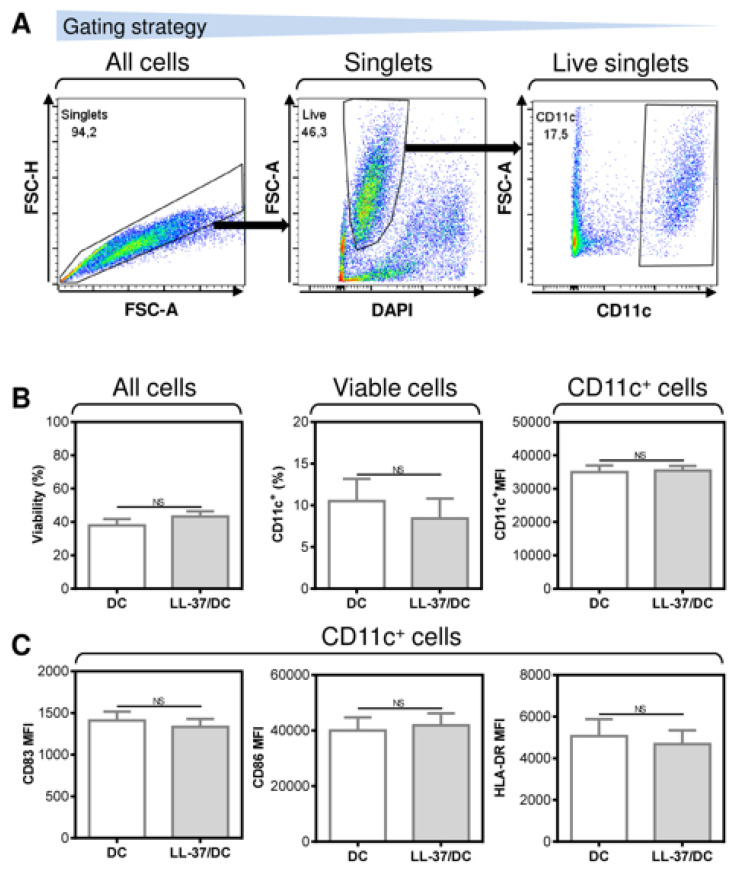
The application of LL-37 during DC production (differentiation, pulsation, and maturation) had no impact on DC viability, yield, or maturation. (**A**) The gating strategy used to analyze flow cytometry data. (**B**) The viability (left panel), DC frequency (CD11c^+^, middle panel), and their CD11c^+^ staining intensities (right panel). (**C**) The staining intensities of the DC maturation markers CD83 (left panel), CD86 (middle panel), and HLA-DR (right panel). In (**B**,**C**), mean + SEM and significances of differences between the group of DC and LL-37/DC samples are indicated (^NS^ *p >* 0.05; *n* = five donors; paired two-tailed Student’s *t*-test).

The enhanced ability of LL-37-treated DCs to promote the enrichment of autologous lymphocytes with T cells and their PD-1-downregulated CD8^+^ subpopulations, including the pulsed antigen-specific ones, indicates that, under the tested conditions, LL-37 could also enhance the level of DC maturation. However, our next analyses revealed that LL-37 treatment had no notable impact on the surface expression of the DC maturation markers in the pulsed and matured DCs (Figure 5). These data, therefore, showed that, under the tested conditions, other mechanisms than the expression levels of the DC maturation markers were responsible for the LL-37-elicited changes in the DC functionality.

### 3.4. LL-37-Treatment of DCs Resulted in the Production of T Cells with Superior Efficacy against Tumor Cells In Vitro

**Figure 6:**
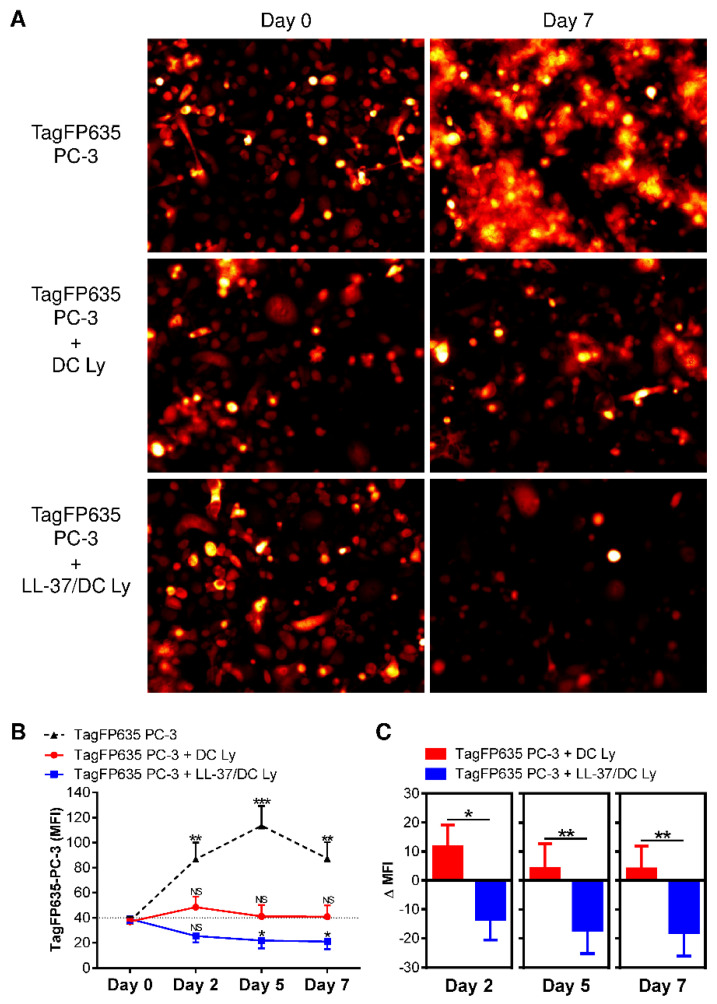
The application of LL-37 during DC production (differentiation, pulsation, and maturation) significantly enhanced the cytotoxic impact of DC-stimulated autologous lymphocytes on cultured tumor cells. (**A**) The 14-day autologous lymphocytes stimulated with DCs (DC Ly) or DCs produced in the presence (differentiation, pulsation, and maturation) of LL-37 (LL-37/DC Ly) were co-cultured with fluorescent TagFP635 PC-3 cells at the ratio of 5:1 (lymphocytes: PC-3 cells) for 7 days. TagFP635 fluorescence in the co-culture was acquired on days 0 (left images) and 7 (right images) of co-culturing. (**B**) The TagFP635 fluorescence in the co-culture was acquired on days 0, 2, 5, and 7 of co-culturing, and the mean fluorescence intensity (MFI) of the acquired images was calculated. The differences in the MFIs of individual days of co-culture for individual samples were statistically evaluated. The data represent mean + SEM or mean—SEM, and the significance of differences among the MFIs are indicated (^NS^ *p >* 0.05, * *p* < 0.05, ** *p* < 0.01, *** *p* < 0.001; *n* = eight donors; repeated measures one-way ANOVA with the Tukey’s post-test). (**C**) The differences between the MFIs before co-culturing (day 0) and at each individual day of co-culturing (days 2, 5, and 7) (ΔMFIs) were calculated for the DC Ly and LL-37/DC Ly samples and statistically evaluated. The data represent means + SEM or means—SEM, and the significance of differences among the ΔMFIs are indicated (* *p* < 0.05, ** *p* < 0.01; *n* = eight donors; paired two-tailed Student’s *t*-test).

The critical feature of DCs for cancer immunotherapy is their ability to induce the expansion of CD8^+^ T cells, which can recognize tumor cells and eliminate them efficiently and sustainably. To evaluate this ability, we co-cultured DC-expanded autologous lymphocytes with fluorescent PC-3 cells (TagFP635 PC-3) [21]. As shown in Figure 6, we confirmed that, without the presence of lymphocytes, the fluorescent TagFP635 PC-3 cells expanded in the cell culture. When these fluorescent tumor cells were co-cultured with the DC-expanded autologous lymphocytes, the expansion of the tumor cell culture was inhibited. However, when LL-37-treated DCs were used to expand autologous lymphocytes, these expanded lymphocytes were not only able to block the tumor cell culture expansion but also cause its contraction. These data showed that autologous lymphocytes expanded with LL-37-treated DCs had a superior anti-tumor cell performance compared to their non-treated counterpart.

## 4. Discussion

This study showed that the design of individual steps of ex vivo DC production could substantially influence DC functionality towards tumor cells. Here, we showed that the previously described modulator of DCs, LL-37, can substantially improve the anti-tumor activity of ex vivo-produced DCs. This enhancement was found to be conditioned by the presence of LL-37 during DC differentiation, pulsation, and maturation, and to lie in the enhanced ability of such LL-37-treated DCs to induce the expansion of autologous CD8^+^ T cells with downregulated PD-1 expression and to enrich the expanded cell culture with CD8^+^ T cells that are reactive to tumor cells expressing antigens used for the pulsation of these DCs. This enhanced ability was then translated into a superior in vitro anti-tumor cell activity of the DC-expanded autologous lymphocytes.

Monocytes from the peripheral blood are largely used to produce DCs for clinical use [26]. The aim of the production is to generate DCs that can efficiently cross-present the DC-loaded (pulsed) tumor antigens and induce an in vivo Th1 response with the subsequent expansion of cytotoxic tumor antigen-specific CD8^+^ T cells [27,28]. This aim was attempted by using different designs of the DC production protocols. These designs concerned the cell culture media composition [29], the monocyte isolation and differentiation [28,30], the form and the way the tumor antigens are loaded into DCs [31,32], and finally, which DC maturation compounds are being used [33,34,35]. This multifactorial complexity of DC production makes finding a robust protocol for the clinical production of DCs with desired functionality extremely difficult. Therefore, there have been multiple attempts to find in vitro potent modulators that could ensure the desired DC functionality. However, to harvest the potential of these modulators, their use needs to be considered with respect to which phase and how these modulators could be used during the DC production. In this study, we confirmed a previous report which demonstrated that LL-37 supplementation into a cell culture medium during DC differentiation promotes the production of human-monocyte-derived DCs with the enhanced potential to induce the expansion of autologous CD8^+^ T cells [17]. However, this enhancement was not strong enough in our experimental settings to attain statistical significance within the tested number of donors. Nevertheless, this enhancement was much higher and already significant when LL-37 was also supplemented during the DC pulsation and maturation. This synergy was not likely caused by enhanced maturation as our data showed no contribution of LL-37 treatment to enhance the expression of the DC maturation markers. This finding is in line with a previous study where LL-37 was found to rather suppress the maturation of DCs, which was elicited via Toll-like receptors (TLRs) [15]. In addition, this described suppression was also associated with impaired DC functionality [15]. Contrasting these findings was a study that showed that the presence of LL-37 during the DC differentiation but not maturation had the opposite impact on the DC functionality [16]. These mutually contrasting findings indeed demonstrated that LL-37 was a strong modulator of the DC functionality, but the outcome of its modulatory impact was largely dependent on when LL-37 was used during the process of DC production [15,16]. Our data added more complexity to these findings as we showed that LL-37 treatment during the whole process of DC production (differentiation, pulsation, and maturation) did not necessarily drive the previously described contrasting impacts of IL-37 against each other but synergized in enhancing the anti-tumor functionality of the produced DCs.

The mechanism through which the LL-37-based DC functionality-promoting synergy works is unknown. However, this mechanism can step into play independent of how LL-37 is used during DC pulsation and maturation. As shown in this study, using UV-irradiated tumor cell lysates allowed us to produce DCs that could then induce at least detectable levels of tumor-cell-reactive CD8^+^ T cells after co-culture with autologous lymphocytes. The supplementation of these UV-irradiated tumor cell lysates with LL-37 and its use for DC pulsation during their maturation was sufficient to significantly enhance not only the overall proportion of CD8^+^ T cells but also the proportion of the tumor-cell-reactive CD8^+^ T cells in the DC-stimulated autologous lymphocytes. These data thus suggest that the cross-presentation of the pulsed tumor cell antigens by DCs was enhanced by LL-37, presumably by forming complexes with nucleic acids and shuttling them across the cell membranes [36]. This mode of action of LL-37 has already been described in previous studies where the formation of LL-37 with immunocomplexes promoted the reactivity of autoantigens via their presentation by plasmacytoid DCs [37]. In addition, the overall promotion of the DC functionality during the pulsation could also be mediated by LL-37 interaction with other immunoreactive compounds, such as cGAMP. This molecule is produced by tumor cells [38], its production is enhanced by UV irradiation [39,40], and its interaction with LL-37 enhances its antiviral immunity [41]. This mechanism could then indicate that it is not the form of the antigen in the cell lysate but the cell lysate itself that conditions the LL-37 modulatory performance towards the DC functionality. However, regardless of the identity of the LL-37-driven mechanisms in the DC production protocol of this study, these mechanisms were able to work together and significantly enhance the functionality of the produced DCs, and this enhanced functionality translated into the in vitro induction of autologous CD8^+^ T cells with a superior potential to act against tumor cells in vitro.

The identity of LL-37 in regulating the immune cells is still elusive. This antimicrobial peptide is expressed in many cell types [42], including epithelial cells [43] and immune cells [42,43], and its impact on the immune system can be either immunosuppressive [44,45] or immunostimulatory [46]. The in vitro data from previous studies indicated that one of the key parameters driving the LL-37 immunomodulatory identity could be the time during which the immune cells were exposed to this peptide. Whereas the acute impact of LL-37 on immature DCs was shown to suppress their maturation and the related functionality [15], chronic exposure to LL-37 during DC development had the opposite effect [16,17]. This acute- vs. chronic-exposure-driven immunomodulatory duality was previously also observed for other molecules, such as stem cell factor [47], IL-33 [48], and IL-6 [49]. In addition, the acute conditioning of human mast cells by preventing their F-actin assembly was shown to even switch their response to stem cell factors from chemotaxis to degranulation [50]. The data of this study further extended these previous findings by showing that the chronic exposure of differentiating monocytes to LL-37 switches the acute response of immature DCs to LL-37 from impaired to enhanced functionality towards CD8^+^ T cell responses. These findings thus further corroborate the concept in the time-dependent conditioning of cells, and their signaling could substantially reprogram the immunomodulatory identity of the molecules that impact them [47,48,49]. In vivo, this identity reprogramming could then define the identity of LL-37 as an immunosuppressant or immunostimulant and thus explain its contrasting roles under different physiological or pathological settings [46,51,52,53,54,55,56,57,58,59,60]. This study showed that in vitro, this time-dependent conditioning concept could be harvested and used as a technological algorithm for boosting the performance of ex vivo-produced immune cells for immunotherapy.

## 5. Conclusions

In this study, we showed that LL-37, with its previously shown ambivalent performance towards the DC functionality, could be incorporated into the DC production protocol in a way in which it could perform synergistically by increasing the functionality of DCs for cancer ACI.

## Data Availability

Not applicable.
